# Epidemiology of *Mycoplasma genitalium* Infections of the Genitourinary Tract Among Attendees of STI Clinic in Hangzhou, China

**DOI:** 10.1002/iid3.70357

**Published:** 2026-02-09

**Authors:** Xinmin Qiu, Jiazhen Shi, Hongqin Gu, Jifeng Liu

**Affiliations:** ^1^ Department of Clinical laboratory Hangzhou Third People's Hospital Hangzhou Zhejiang People's Republic of China; ^2^ Department of Dermatology Hangzhou Third People's Hospital Hangzhou Zhejiang People's Republic of China

**Keywords:** co‐infection, epidemiology, mycoplasma genitalium, symptoms

## Abstract

**Objective:**

This study aimed to investigate the prevalence, associated clinical manifestations, and co‐infection patterns of *Mycoplasma genitalium* (MG) infection among patients attending Venereal Disease, Urology, and Gynecology clinics in Hangzhou, China. The findings provide foundational evidence to guide sexually transmitted infection (STI) screening in patients with genitourinary tract conditions.

**Methods:**

Between January 2020 and December 2023, 12,934 outpatients from Hangzhou Third People's Hospital were tested for MG using isothermal amplification targeting the 16S rRNA gene. Concurrent testing for *Neisseria gonorrhoeae* (NG), *Chlamydia trachomatis* (CT), and *Ureaplasma urealyticum* (UU) was performed.

**Results:**

Of these 12,934 patients, 589 tested positive for MG, yielding an overall prevalence of 4.55% (589/12,934). Prevalence was significantly higher in males (416/7915, 5.26%) than in females (173/5,019, 3.44%). Significant age‐related differences were observed (*p* < 0.001), with the highest prevalence in patients ≤ 20 years 7.83% and the lowest in those > 60 years 0.57%. Of 416 MG‐positive males, 295 (70.91%) exhibited symptoms of urethritis, prostatitis, and epididymitis. whereas 49.71% (86/173) of MG‐positive females exhibited vaginitis, cervicitis, or pelvic inflammatory disease (*p* < 0.001). Symptomatic infection rates were significantly higher in males than in females. Co‐infections were found in 44.31% (261/589) of MG‐positive patients, with MG + UU co‐infection being the most frequent 22.92% (135/589).

**Conclusions:**

MG infection prevalence in Hangzhou is substantial, with significantly higher rates among males and young adults (≤ 20 years). Females exhibit markedly lower symptomatic infection rates. MG demonstrates frequent co‐infection, predominantly with UU, underscoring the necessity of multipathogen testing to prevent missed diagnoses.

## 1 Introduction

Mycoplasma comprises a group of bacteria lacking cell walls, exhibiting pronounced polymorphism and the capacity for filterable propagation and cultivation in artificial media [1]. As the smallest known prokaryotic organisms (0.1–0.3 µm), mycoplasmas are widely distributed in humans and animals, with 16 species potentially isolated from humans [2]. Key species causing human genitourinary infections include *Mycoplasma hominis* (MH), *Ureaplasma urealyticum* (UU), *Mycoplasma genitalium* (MG), *Mycoplasma penetrans*, *Mycoplasma fermentans*, and *Microureaplasma*. Recently, it has been suggested that *Mycoplasma fermentans* develops bacterial vaginosis [3].

Current guidelines recommend a syndromic approach for suspected sexually transmitted infections (STIs), with routine testing typically including *Chlamydia trachomatis* (CT), *Neisseria gonorrhoeae* (NG), and in some settings, *Trichomonas vaginalis* (TV) [4]. Due to increasing macrolide resistance, MG testing is now advised for persistent or recurrent urethritis/cervicitis. First‐line treatment involves doxycycline (pre‐treatment) followed by azithromycin or moxifloxacin, with test‐of‐cure (TOC) recommended 3–4 weeks post‐treatment given frequent therapeutic failures [5, 6].

MG causes urethritis, cervicitis, proctitis, pelvic inflammatory disease (PID), prostatitis, and epididymitis, and is associated with adverse pregnancy outcomes [7]. Additionally, MG enhances HIV acquisition and transmission [8, 9]. First isolated from men with non‐gonococcal urethritis (NGU) by Tully et al. in 1981 [10], MG possesses a minimal genome (~580 kb) [11], making it the smallest free‐living prokaryote colonizing humans. The diagnosis of MG infection mainly relies on pathogen examination. In vitro cultivation is challenging, time‐consuming, and unsuitable for clinical routine. Nucleic acid amplification tests, which detect MG‐specific DNA or RNA in clinical specimens [12], offer high sensitivity and specificity and represent the current gold standard for MG diagnosis.

The World Health Organization (WHO) global estimates of curable STIs lack data for MG, likely due to insufficient epidemiological evidence from many regions of the world. As MG infections are not notifiable in China, systematic surveillance data are similarly unavailable. Consequently, precise MG prevalence remains undefined, with reported rates varying significantly across populations, geographical regions, and anatomical sites. In Britain and Australia, incidence is 1.07 per 100 person‐years [13], contrasting with rates as high as 33.4 per 100 person‐years among sexually active women with vaginitis in Kenya and the United States [14]. A Chinese meta‐analysis revealed an MG prevalence of 0.94% in the general population undergoing health examinations, rising to 7.32–15.22% in STI clinics, gynecology, and urology departments. Among high‐risk groups—men who have sex with men (MSM), female sex workers (FSWs), and HIV‐positive individuals—prevalence reaches 9.70%, 13.49%, and 20.46%, respectively [15].

Despite these global and national estimates, significant gaps persist in regional MG epidemiology. Hangzhou, a metropolitan hub exceeding 12 million residents with high population mobility, represents a distinct epidemiological setting where MG transmission dynamics may differ substantially from Western populations and other Chinese regions. Furthermore, the city's subtropical monsoon climate, characterized by high humidity, may further influence pathogen persistence and transmission patterns. However, no comprehensive MG prevalence data currently exist for this strategically vital Yangtze River Delta region. To address this critical knowledge gap, we conducted MG, UU, NG, and CT testing among 12,934 patients with suspected genitourinary infections attending dermatology (venereology), urology, and gynecology clinics in The Third People's Hospital of Hangzhou, with stratified analysis by age, gender, and calendar year to establish local epidemiological baselines.

## 2 Patients and Methods

### 2.1 Patients

Between January 2020 and December 2023, 12,934 patients (7915 males, 5019 females) presenting with suspected genitourinary tract infections were enrolled from the Dermatology (Venereology), Urology, and Gynecology departments of Hangzhou Third People's Hospital. Male patients aged 18–67 years (average: 39.20 ± 17.54) and females aged 18–76 years (average: 36.55 ± 14.16) were stratified into six age groups: ≤ 20, 21–30, 31–40, 41–50, 51–60, and >60 years.

The inclusion criteria were as follows: 1. Patients displaying symptoms consistent with urogenital tract infections. For males, these symptoms included: urethral discharge (purulent or mucinous), dysuria, increased frequency of urination, urgency, urethral pruritus or burning sensation, testicular or epididymal pain or swelling, and perineal discomfort. For females, symptoms comprised: an increase in vaginal discharge (with abnormal color and odor), pruritus, burning pain or redness of the vulva, discomfort during sexual intercourse or bleeding post‐coitus, lower abdominal pain or pelvic discomfort, dysuria, or frequent urination. Additionally, symptoms such as perineal ulcers and enlarged inguinal lymph nodes were noted. 2. Asymptomatic individuals who had no evident clinical symptoms but were concerned about potential infections due to high‐risk sexual activities, as well as partners who had been diagnosed with MG infection. 3. Participants must be between 18 and 80 years, whether married or unmarried, but with a history of sexual activity. Exclusion criteria included: 1. Individuals with no history of sexual activity. 2. Those who have used antibiotics in the 2 weeks prior to the study. 3. Pregnant and lactating women.

### 2.2 Methods

#### 2.2.1 Sample Preparation

Urethral (male) or endocervical (female) swabs were inserted 1–2 cm, rotated three times clockwise, retained for 15 s, and withdrawn. Swabs were immersed in 1 mL sterile saline and expressed against the collection tube walls to release absorbed fluid. First‐void urine (≥2 h urinary retention) or initial morning void specimens were collected. All specimens were immediately mixed 1:1 with RNA‐stabilizing solution (kit‐provided) for preservation.

#### 2.2.2 RNA Detection of MG, UU, CT, and NG

Clinical specimens (urine, urethral/cervical swabs) were tested using the Acid Detection Kit (RNA Isothermal Amplification Assay; Shanghai Rendu Biological Technology Co. Ltd.), which targets the 16S rRNA gene of *Mycoplasma genitalium* (MG), *Chlamydia trachomatis* (CT), *Neisseria gonorrhoeae* (NG), and *Ureaplasma urealyticum* (UU). In accordance with the manufacturer's instructions, a total of 400 μL of preserved samples underwent nucleic acid extraction via magnetic bead purification, involving lysis at 60°C and two subsequent wash steps. This was followed by target‐specific amplification, which consisted of 40 cycles at 42°C, with real‐time fluorescence detection conducted at the end of each cycle. Results were considered positive if the cycle threshold (Ct) was ≤ 35. Each run was validated with internal controls.

### 2.3 Statistical Analyses

All analyses were performed using SPSS Statistics software for Windows, version 24.5 (IBM Corp). Measurement data were analyzed using the Chi‐square test, and *t*‐test was used for counting data, with *p* < 0.05 as the cut‐off for significance.

## 3 Results

### 3.1 Overall Infection Rates

Of the 12,934 patients in this study, 589 (4.55%) cases tested positive for MG. The positivity rate was significantly higher in males (5.26%; 416/7915) compared to females (3.44%; 173/5019), with *χ* ^2^ = 23.123, *p* < 0.001. In addition, 573 cases tested positive for CT, including 509 males and 64 females, demonstrating a significantly higher prevalence in males (*χ* ^2^ = 192.94, *p* < 0.001). Additionally, 631 cases were found positive for NG, with 613 males and 18 females, which also showed a significantly higher occurrence in males (*χ* ^2^ = 360.68, *p* < 0.001). On the contrary, there were 3720 cases that tested positive solely for UU, consisting of 1377 males and 2343 females. This group exhibited a significantly higher positivity rate in females (*χ* ^2^ = 1285.7, *p* < 0.001).

### 3.2 Comparison of Infection Rates Among Age Groups

Of the 12,934 patients evaluated in this study, MG prevalence varied significantly across age groups (*χ* ^2^ = 96.745, *p* < 0.001), with rates declining progressively: ≤ 20 years: 7.83%, 21–30 years: 6.01%, 31–40 years: 4.96%, 41–50 years: 3.43%, 51–60 years: 2.74%. The highest and lowest prevalence occurred in the ≤ 20‐year (7.83%) and >60‐year (0.57%) cohorts, respectively. When examining the infection rates by sex, males exhibited rates of 5.29%, 6.86%, 6.25%, 4.28%, 4.40%, and 0.76% across the age groups, showing significant differences (*χ* ^2^ = 57.074, *p* < 0.001). In contrast, the rates for females were 11.42%, 4.93%, 2.73%, 2.04%, 0.44%, and 0%, also indicating significant differences among the groups (*χ* ^2^ = 74.211, *p* < 0.001). Notably, the highest infection rate among males was found in the 21–30 age group (6.86%), whereas females exhibited the highest rate in the ≤ 20 age group (11.42%). Both genders presented the lowest positivity rates in those aged > 60 years, at 0.76% for males and 0% for females (Table 1).

**TABLE 1:** Gender‐specific differences in MG infection rates.

Age	Males (*n* = 7915), %(*n*/*n*)	Females (*n* = 5019), %(*n*/*n*)	Total (*n* = 12,934), %(*n*/*n*)
≤ 20	5.29 (19/359)	11.42 (29/254)	7.83 (48/613)
21–30	6.86 (153/2231)	4.93 (87/1765)	6.01 (240/3996)
31–40	6.25 (151/2415)	2.73 (38/1394)	4.96 (189/3809)
41–50	4.28 (58/1355)	2.04 (17/832)	3.43 (75/2187)
51–60	4.40 (28/637)	0.44 (2/459)	2.74 (30/1096)
> 60	0.76 (7/918)	0 (0/315)	0.57 (7/1233)
*χ* ^2^	57.074	74.211	96.745
*p*	< 0.001	< 0.001	< 0.001

### 3.3 Comparison of Annual MG Infection Rates

**FIGURE 1:**
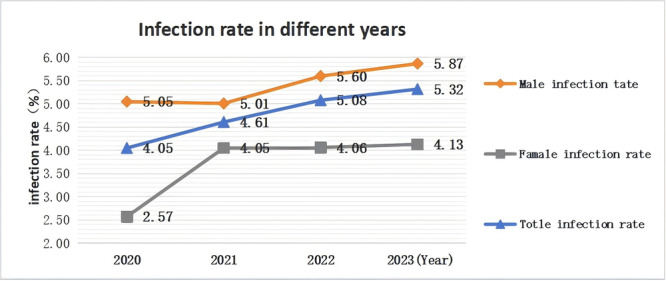
Comparison of infection rates during different years.

The overall MG prevalence rates for 2020–2023 were 4.05%, 4.61%, 5.08%, and 5.32%, respectively, demonstrating a non‐significant increasing trend (*χ* ^2^ = 6.690, *p* = 0.072). Respective infection rates for males in these 4 years were 5.05%, 5.01%, 5.60%, and 5.87%, showing no significant differences across these years (*χ* ^2^ = 2.083, *p* = 0.564). Conversely, the rates for females were 2.57%, 4.05%, 4.06%, and 4.13%, respectively, with an upward trend and significant differences in these rates across these years (*χ* ^2^ = 8.296, *p* = 0.040) (Table 2, Figure 1).

**TABLE 2:** MG infection rates during different years.

Year	Males (*n* = 7915), %(*n*/*n*)	Females (*n* = 5019), %(*n*/*n*)	Total (*n* = 12934), %(*n*/*n*)
2020	5.05 (158/3130)	2.57 (54/2099)	4.05 (212/5229)
2021	5.01 (125/2495)	4.05 (74/1826)	4.61 (199/4321)
2022	5.6 (30/536)	4.06 (11/271)	5.08 (41/807)
2023	5.87 (103/1754)	4.13 (34/823)	5.32 (137/2577)
*χ* ^2^	2.038	8.296	6.990
*p*	0.564	0.040	0.072

### 3.4 Comparison of MG Infection Rates Among Male Patients With Different Symptoms

Among MG‐positive males, urethritis was the predominant clinical manifestation, followed by prostatitis and/or epididymitis, and HIV infection. MG prevalence was significantly higher in symptomatic patients versus asymptomatic individuals. Among 7915 male patients evaluated, 3202 (40.5%) were asymptomatic and 4713 (59.5%) presented with symptoms. The prevalence of MG infection was significantly higher in symptomatic patients, with a rate of 8.3% (389/4713), compared to just 0.8% (27/3202) in asymptomatic individuals (*p* < 0.001). These findings indicate that the MG infection rate is lower among patients who do not exhibit the aforementioned symptoms, as summarized in Table 3.

**TABLE 3:** Comparison of MG infection rates among males with different symptoms.

Symptom	Number	MG positive	MG negative	*χ* ^2^	*p*
Number	7915	416	7499	/	/
Urethritis					
Yes	2127	217	1910	81.820	< 0.001
No	5788	199	5589		
Prostatitis/Epididymitis					
Yes	415	78	337	161.227	< 0.001
No	7500	338	7162		
Condyloma acuminata					
Yes	907	19	888	20.555	< 0.001
No	7008	397	6611		
Syphilis					
Yes	803	21	782	12.514	< 0.001
No	7112	395	6717		
Genital herpes					
Yes	457	18	439	1.69	0.194
No	7458	398	7060		
HIV					
Yes	4	1	3	3.133	0.077
No	7911	415	7496		
Asymptomatic*					
Yes	3202	27	3175	210.268	< 0.001
No	4713	389	4324		

* Asymptomatic: individuals with no genitourinary symptoms but tested due to high‐risk sexual behavior or partner infection.

### 3.5 Comparison of MG Infection Rates Among Females with Different Symptoms

Among female patients who tested positive for MG, vaginitis and cervicitis were identified as the most prevalent symptoms, followed closely by pelvic inflammatory disease. The positivity rate for MG in these patients was significantly higher compared to those without the aforementioned symptoms. Among patients diagnosed with condyloma acuminata, syphilis, and herpes, the MG infection rate was lower than that of patients without syphilis and genital herpes, indicating that MG may also be related to ulcerative diseases of the external genitalia. In a cohort of 5,019 female patients, 1112 (22.2%) were asymptomatic, with 52 testing positive for MG (4.7%). Conversely, among the 3907 symptomatic females (77.8%), there were 121 MG‐positive cases (3.1%). This difference in prevalence was statistically significant (*p* = 0.011), as shown in Table 4.

**TABLE 4:** Comparison of MG positivity rates among females with different symptoms.

Symptom	Number	MG positive	MG negative	*χ* ^2^	*p*
Number	5019	173	4846	/	/
Vaginitis/cervicitis					
Yes	1627	61	1566	0.661	0.416
No	3392	112	3280		
Pelvic inflammation					
Yes	245	25	220	35.338	< 0.001
No	4774	148	4626		
Condyloma acuminata					
Yes	817	32	785	1.528	0.221
No	4202	141	4061		
Syphilis					
Yes	698	9	689	11.34	< 0.001
No	4321	164	4157		
Genital herpes					
Yes	519	4	515	12.457	< 0.001
No	4500	169	4331		
HIV					
Yes	1	0	1	0.036	0.850
No	5018	173	4845		
Asymptomatic*					
Yes	1112	52	1060	6.487	0.011
No	3907	121	3786		

* Asymptomatic: individuals with no genitourinary symptoms but tested due to high‐risk sexual behavior or partner infection.

### 3.6 Gender‐Related Differences in Clinical Symptoms

Of the 416 male patients positive for MG, 295 (70.91%) exhibited symptoms of urethritis, prostatitis, and epididymitis. Of the 173 female patients positive for MG, 86 (49.71%) exhibited symptoms of vaginitis, cervicitis, and pelvic inflammatory disease. The proportion of symptomatic male patients was significantly higher than that of female patients (*χ* ^2^ = 24.046, *p* < 0.001), suggesting that females are more susceptible to asymptomatic infections (Table 5).

**TABLE 5:** Comparison of inflammation of the urinary and reproductive tracts between male and female MG‐infected patients.

	Number	With inflammation of the urinary and reproductive tracts	Without inflammation of the urinary and reproductive tracts	*χ* ^2^	*p*
	589	374	215		
Males	416	295	121	24.046	< 0.001
Females	173	86	87		

### 3.7 Co‐Infection Prevalence Among MG Patients

Of the 589 patients positive for MG, 261 (44.31%) were mono‐infected with MG. The most common co‐infections among patients in this study were MG and UU (MG + UU), accounting for 22.92% (135/589) of MG‐positive patients, followed by MG + CT + UU, MG + CT, MG + NG, MG + NG + UU, MG + CT + NG + UU, and MG + CT + NG with respective prevalence rates of 16.81% (99/589), 6.62% (39/589), 3.90% (23/589), 2.55% (15/589), 1.70% (10/589), and 1.19% (7/589). Of the 416 MG‐positive males, 234 were mono‐infected with MG (56.25%), with this rate being significantly higher than the rate of mono‐infection among females (15.61%; 27/173) (*χ* ^2^ = 81.792, *p* < 0.001). Among males, the most common co‐infection was MG + UU, accounting for 17.07% (71/416) of MG‐positive males, followed by MG + CT, MG + CT + UU, MG + NG, MG + NG + UU, MG + CT + NG, MG + CT + NG + UU with respective prevalence rates of 8.41% (35/416), 7.45% (31/416), 5.53% (23/416), 2.64% (11/416), 1.44% (6/416), and 1.20% (5/416). Of the 173 MG‐positive females, 27 were mono‐infected with MG (15.61%). Co‐infections were more common, with MG + CT + UU and MG + UU respectively accounting for 39.30% (68/173) and 36.99% (64/173) of these MG‐positive cases, followed by MG + CT + NG + UU, MG + NG + UU, MG + CT, and MG + CT + NG, with respective prevalence rates of 2.89% (5/173), 2.31% (4/173), 2.31% (4/173), and 0.58% (1/173) (Table 6).

**TABLE 6:** MG co‐infection with CT, NG, and UU.

Coinfection with other pathogens	Male (*n* = 416), %(*n*/*n*)	Female (*n* = 173), %(*n*/*n*)	Total (*n* = 589), %(*n*/*n*)
MG + CT	8.41 (35/416)	2.31 (4/173)	6.62 (39/589)
MG + NG	5.53 (23/416)	0 (0)	3.90 (23/589)
MG + UU	17.07 (71/416)	36.99 (64/173)	22.92 (135/589)
MG + CT + UU	7.45 (31/416)	39.30 (68/173)	16.81 (99/589)
MG + NG + UU	2.64 (11/416)	2.31 (4/173)	2.55 (15/589)
MG + CT + NG	1.44 (6/416)	0.58 (1/173)	1.19 (7/589)
MG + CT + NG + UU	1.20 (5/416)	2.89 (5/173)	1.70 (10/589)
Only MG	56.25 (234/416)	15.61 (27/173)	44.31 (261/589)

## 4 Discussion

MG is an important pathogen that is responsible for high rates of STI, accounting for 15–20% of NGU cases [4]. In females, MG infections are associated with lower reproductive tract infections, cervical miscarriage, and infertility [16]. The common diagnostic approach, nucleic acid amplification testing, offers high levels of sensitivity and specificity, such that it is the most widely used method for detecting MG [17]. As such, an SAT approach was used to test 12,934 patients for MG, yielding an overall positivity rate of 4.55% which was higher than the 3.64% rate previously reported in Hunan Province [18], although it was lower than the 6.48% rate in Taizhou city [19], 7.95% in Nanjing city [20], and 16.5% in Shandong Province [21]. Additionally, the observed positivity rate aligned closely with the rates of 4.02% and 4.4% reported in Zhoushan City [22] and Guangzhou City [23], respectively, indicating that MG infection rates exhibit notable regional variations across China.

Our gender‐stratified analysis revealed significantly higher MG prevalence in males (5.26%) versus females (3.44%), consistent with Lu et al. [19] but contrasting with Zhang et al. [24]. These differences may be attributable to population‐specific characteristics or differences in patient sexual activity. We further observed a pronounced inverse relationship between age and MG prevalence, with peak infection rates in the ≤ 20‐year cohort (7.83%) and minimal rates in >60‐year patients (0.57%). For both males and females, gender‐specific peaks occurred at 21–30 years for males (6.86%) and ≤ 20 years for females (11.42%), respectively, consistent with results reported by Zhang et al. [25]. This age clustering supports the established association between MG transmission and sexual activity [26], particularly given the natural decline in sexual frequency with aging and the sexual transmission route of genitourinary pathogens. The high prevalence among females ≤ 20 years may reflect physiological susceptibility and inadequate sexual health knowledge in this demographic. These findings underscore the imperative to enhance targeted sexual health education and protective behavior promotion for young women.

From 2020 to 2023, MG prevalence demonstrated a gradual increase, potentially attributable to post‐COVID‐19 pandemic factors, including resumed population mobility and reopening of bathing/entertainment venues. MG frequently co‐occurs with other STI pathogens, NG and CT [27, 28]. Among our 589 MG‐positive patients, monoinfection prevalence (44.31%) aligned with Braam et al. [29]. The most common co‐infection in this population was MG + UU (22.92%). Notably, monoinfection was significantly higher in males (56.25%, 234/416) than in females (15.61%). Among the 173 female MG patients, the most common co‐infections were MG + CT + UU (39.30%) and MG + UU (36.99%). Among the 416 male MG patients in this study cohort, 70.91% exhibited symptoms, with this rate being significantly higher than the 45.66% of females affected by vaginitis, cervicitis, and pelvic inflammatory disease, suggesting that females are more susceptible to asymptomatic infections and co‐infections [30]. Asymptomatic women may delay seeking medical care, potentially facilitating ongoing transmission. To mitigate missed diagnoses, clinicians should implement multiplex pathogen testing (MG, CT, NG) for suspected urogenital infections, particularly in female patients. Given MG's high pathogenicity and frequent co‐infections with organisms like UU, for patients with symptoms of urinary and reproductive tract infections, especially young and middle‐aged men and women, it is recommended to undergo MG, NG, UU, and CT examinations. While European guidelines do not recommend MG testing for asymptomatic patients [31], it is important to consider the serious consequences that can arise from MG infections, such as infertility and miscarriage. Given that women are often asymptomatic, young sexually active individuals, particularly those with multiple sexual partners and high‐risk behaviors, should be encouraged to undergo MG testing. Additionally, asymptomatic patients over the age of 60 may have a lower necessity for testing. Although screening for asymptomatic carriers may increase the consumption of medical resources, it needs to be determined based on the actual situation of the patient. With advancements in molecular biology detection technologies, including SAT, urine is now a viable sample source that patients can easily collect themselves. and our SAT‐based self‐sampling approach demonstrated dual advantages of accuracy and acceptability. Validation studies showed urine SAT achieved 98.77% sensitivity and 94.31% specificity for NG detection compared to culture (*n* = 204), with 98.53% concordance between urine and swab samples—supporting its reliability as a non‐invasive alternative [32]. Crucially, this method improved patient compliance, with 89% of participants (especially young women) preferring self‐collection over clinician‐obtained swabs(date was not shown). Meanwhile, doctors do not have to sample each patient one by one during the busy consultation process, further enhancing its applicability in high‐throughput screening in the clinical environment.

This study has several limitations. First, it is a single‐center analysis that specifically examines the co‐infection status of MG‐positive patients in the Hangzhou area. Second, patient treatment status was not analyzed. Third, no efforts were made to detect any resistance genes in MG isolates from these patients. It is important to note that MH was excluded from this study due to its absence in routine clinical testing during the study period. While MH's role in genitourinary infections remains debated [33, 34], its potential interactions with MG/UU warrant future investigation. To address these limitations in future research, we plan to conduct multi‐center studies that will provide a more comprehensive understanding of the epidemiology of MG infections and co‐infections across China. Furthermore, we will analyze the therapeutic regimens utilized for patients in China diagnosed with MG and conduct tests to clarify the distribution of resistance genes and co‐infection rates concerning MG in the country.

## 5 Conclusions

The findings of this study highlight the high prevalence of MG in Hangzhou, China, providing important epidemiological insights. The data indicate that infection rates are correlated with both age and gender, with the highest incidence observed among sexually active young adults. Women are prone to co‐infections, such as MG complicated with UU, and the prevalence of asymptomatic infections is also higher in women compared to men. Asymptomatic individuals harboring MG pose a risk of being potential transmission sources. To prevent missed diagnoses, it is necessary to carry out MG, UU, NG, and CT detection for patients with multiple sexual partners or those leading active sexual lives who are suspected of having genitourinary tract infections. Prompt treatment of those diagnosed as positive can significantly curtail the transmission of MG. Moreover, future research is crucial for establishing multi‐center monitoring points, evaluating treatment efficacy, and investigating the prevalence of drug resistance genes. These initiatives will lay a robust scientific groundwork for developing effective prevention and treatment strategies for MG infections.

## Author Contributions

**Xinmin Qiu:** investigation, writing – original draft, resources, project administration, data curation. **Jiazhen Shi:** data curation, resources, formal analysis, conceptualization, writing – original draft. **Hongqin Gu:** Data curation, resources, formal analysis, writing – original draft, investigation. **Jifeng Liu:** writing – review and editing, formal analysis, resources, investigation, methodology.

## Ethics Statement

The experimental protocol was established, according to the ethical guidelines of the Helsinki Declaration and was approved by the Human Ethics Committee of Hangzhou Third People's Hospital (Approval Number: 2024KA002). Given the retrospective nature of the study and the use of anonymized data, the Ethics Committee waived the requirement for informed consent.

## Conflicts of Interest

The authors declare no conflicts of interest.

## Data Availability

The data that support the findings of this study are available on request from the corresponding author. The data are not publicly available due to privacy or ethical restrictions.
